# The Influence of Dark Triad Traits on Non-Suicidal Self-Injury Among Left-Behind Adolescents: A Moderated Mediation Model

**DOI:** 10.3390/bs16010137

**Published:** 2026-01-18

**Authors:** Jiale Wang, Tonglin Jin

**Affiliations:** 1 School of Psychology, Inner Mongolia Normal University, Hohhot 010022, China; 18698403733@163.com; 2 Mental Health Education Research and Service Base, Key Research Base of Humanities and Social Science in Inner Mongolia Colleges and Universities, Hohhot 010022, China

**Keywords:** dark triad traits, non-suicidal self-injury, negative life events, depression, adolescents

## Abstract

This study explores the relationship model among the dark triad traits, non-suicidal self-injury (NSSI) behavior in adolescents, negative life events, and depression. A moderated mediation model was tested among 224 middle school students with left-behind experience in Inner Monolgia. These students were surveyed using the Dirty Dozen Dark Triad Measure, the Adolescent Self-Harm scale, the Adolescent Self—Rating Life Events Checklist, and the Center for Epidemiologic Studies Depression Scale. The dark triad traits had a significant positive predictive effect on NSSI behavior among adolescents with left-behind experience and indirectly influenced NSSI behavior through negative life events. The second half of the mediation path of “the dark triad traits → negative life events → NSSI behavior” was moderated by depression. The influence of the dark triad traits on NSSI behavior is exerted through negative life events, and the relationship between negative life events and NSSI behavior is moderated by depression.

## 1. Introduction

Non-suicidal self-injury (NSSI) refers to the deliberate, repeated destruction of one’s own body tissue without suicidal intent, in a manner not socially sanctioned. Common forms include cutting, hitting, burning, banging, and scratching (Zhang et al., 2014). In recent years, NSSI has been globally recognized as a major public health concern among adolescents (Y. Li et al., 2025). International studies indicate that the lifetime prevalence of NSSI in adolescents ranges between 17% and 60% (Brown & Plener, 2017). The Chinese research has found that the most common forms of NSSI among adolescents include hair-pulling, followed by cutting the skin with glass or knives, with 68.2% of those engaging in NSSI using two or more self-harm methods (Jiang et al., 2022). As a risk factor for suicidal mortality, NSSI not only severely jeopardizes adolescents’ physical and mental health (Tang et al., 2023), but also impairs emotional functioning, leading to declines in interpersonal relationships and academic performance (Xian, 2023). In the sociocultural context of China, a particularly noteworthy high-risk group is adolescents with left-behind experiences. They refer to minors under the age of 16 whose parents are both migrant workers, or whose parents are migrant workers with one parent lacking the capacity to provide guardianship (Fan et al., 2023). Research indicates that the risk of NSSI in this group is significantly higher than that among non-left-behind adolescents. For instance, Qian et al. (2024), in a survey of 223 adolescents exhibiting NSSI behaviors, found that left-behind adolescents were 2.41 times more likely to engage in NSSI compared to their non-left-behind peers. Due to prolonged parental separation and perceived lack of parental care, left-behind adolescents generally exhibit weaker psychological resilience and face greater challenges in psychological adaptation (Fan et al., 2018), which exposes this group to a heightened risk of non-suicidal self-injury and underscores the urgent need for attention and intervention regarding mental health.

The dark triad traits refer to a cluster of antisocial personality traits comprising Machiavellianism, narcissism, and psychopathy (Bertl et al., 2017). Although these three personalities have distinct structural characteristics, they share common features such as high aggressiveness, low agreeableness, and emotional coldness. Research indicates that dark triad traits are associated with various adverse psychological and physical health outcomes. Notably, individuals exposed to more adverse childhood environment tend to exhibit higher levels of dark triad traits, which correlate with more frequent and severe self-injurious behaviors (Xian, 2023; Sai et al., 2020). Furthermore, according to the Dual-Harm Cognitive-Affective Model (Shafti et al., 2021), dual-harm behaviors (self-injury and aggression) result from the interaction between distal and proximal factors. Under the influence of distal factors (e.g., biological predispositions and adverse environments), individuals may develop personality traits that predispose them to harmful behaviors. Subsequently, under the influence of proximal factors (e.g., cognitive and emotional processes), these pre-existing personality traits increase the likelihood of dual-harm behaviors, including self-injury and aggression. Based on this theoretical framework, it can be inferred that the dark triad traits serve as an antecedent variable influencing non-suicidal self-injury. In addition, Relevant research indicates that adolescents with experiences of being left behind may develop heightened sensitivity to negative interpersonal patterns triggered by dark triad traits (such as manipulation and emotional coldness) due to parental separation and emotional neglect during their upbringing. This sensitivity may manifest as a distinct pathway to non-suicidal self-injury (NSSI) risk (Zheng et al., 2025). However, prior research has merely examined the influence mechanism of the “dark triad traits” on “non-suicidal self-injury” without specifically considering this group (Mansouri et al., 2023). Consequently, this paper delves into the influence mechanisms of the dark triad traits on non-suicidal self-injury among adolescents with left-behind experiences—a distinct and under-researched cohort. This investigation not only illuminates the unique psychopathological mechanisms underlying non-suicidal self-injury in this group but also provides empirical support for theories concerning non-suicidal self-injury. Given these findings, investigating the influencing factors and underlying mechanisms of NSSI among left-behind adolescents and developing effective interventions is crucial for fostering their healthy development.

Moreover, adolescents experiencing a greater number of negative life events exhibit increased frequency of non-suicidal self-injury behaviours, with such events directly predicting an individual’s non-suicidal self-harm (Yu, 2022; He et al., 2025). negative events life events denote unpleasant occurrences that elicit negative emotional experiences, inducing feelings of unease, pessimism, anxiety, and other distressing affective states that propel individuals towards negative emotional trajectories. Among adolescents, prevalent negative life events include academic pressure, interpersonal relationship difficulties, and early traumatic experiences such as abuse, neglect, or bullying (Tang et al., 2023). These events may all serve as potential precursors to non-suicidal self-injury. The Stress-Vulnerability Model provides a framework for understanding self-injury among adolescents with left-behind experiences, emphasising the negative impact of the interaction between trait vulnerability and environmental stress on adolescent behaviour (Mann et al., 1999; Zhao & Liu, 2025). Specifically, adolescents with dark triad traits who have experienced being left behind exhibit inherent cognitive-emotional patterns that constitute a key determinant of individual vulnerability. This vulnerability, in turn, systematically influences their interactions with the environment. They are more likely to perceive everyday interpersonal friction or academic challenges as severe ‘negative life events’ and become entrenched in more intense negative emotions (Han et al., 2021). Based on this reasoning, the dark triad traits may indirectly elevate the risk of non-suicidal self-injury (NSSI) by shaping and amplifying the negative life events experienced by individuals. Negative life events are thus posited as a mediating factor linking dark triad traits to behavioural outcomes. Accordingly, Hypothesis H2 is proposed: Negative life events mediate the relationship between the dark triad traits and non-suicidal self-injury in adolescents with a history of left-behind experiences.

Concurrently, depression—a prevalent mental health issue among adolescents with potentially far-reaching negative consequences—poses a grave threat to their physical and psychological wellbeing (Xiao et al., 2024). Research indicates that depression amplifies the direct effect of dark triad traits on non-suicidal self-injury (Shang et al., 2022), meaning it impairs individuals’ emotional regulation abilities, making the aggressive impulses generated by dark triad traits more likely to be directed inward. Compared to adolescents without left-behind experiences, those with such backgrounds face heightened depression risks and consequently exhibit weaker emotional regulation capacities. Under these circumstances, they are more likely to internalise aggression impulses induced by dark triad traits, manifesting as non-suicidal self-injury. Moreover, depression intensifies the association between dark triad traits and negative life events. Cognitive theories of depression posit that cognitive distortions are key contributors to depressive onset. Depression may impair cognitive and social functioning, thereby amplifying the tendency of individuals with dark triad traits to actively or passively encounter increased interpersonal conflicts and negative events (Beck, 1979; Bonfá-Araujo et al., 2023). According to the Frustration-Aggression Theory, individuals experiencing setbacks are highly likely to exhibit aggressive behaviour (Qu et al., 2012; Wu et al., 2023). Specifically, when individuals develop negative emotions due to setbacks such as negative life events, and these emotions cannot be vented externally, they are more likely to engage in non-suicidal self-injury. In summary, this study focuses on adolescents with left-behind experiences from middle schools in Inner Mongolia, grounded in the Dual-Harm Cognitive-Affective Model, the Stress-Vulnerability Model, and the Frustration-Aggression Theory, this study aims to investigate: (1) the impact of the dark triad traits on non-suicidal self-injury (NSSI) among left-behind adolescents, (2) the mediating role of negative life events, and (3) the moderating effect of depression in this relationship. Meanwhile, this paper puts forward three hypotheses.

**H1.**  *The dark triad traits significantly and positively predicts NSSI among adolescents with left-behind experiences.*

**H2.**  *Negative life events mediate the relationship between the dark triad traits and NSSI among adolescents with left-behind experiences.*

**H3.**  *Depression moderates the direct path of the dark triad traits influencing non-suicidal self-injury behaviour among left-behind adolescents. Furthermore, the dark triad traits mediates the path from negative life events to non-suicidal self-injury behaviour, specifically the first half (dark triad traits → negative life events) and the second half (negative life events → non-suicidal self-injury behaviour).*

## 2. Materials and Methods

### 2.1. Participants

Using convenience sampling, we recruited 7th–9th grade students with left-behind experiences from three middle schools in Inner Mongolia for questionnaire surveys. Inclusion Criteria: A. Adolescents (under 16 years) with ≥3 months’ cumulative experience of parental migration (one or both parents) for employment purposes. B. Absence of pre-existing or comorbid psychopathological conditions (as confirmed by clinical evaluation). Exclusion Criteria: A. Adolescents without documented parental migration history meeting the above duration threshold. B. Presence of any pre-existing or comorbid psychiatric diagnosis (based on DSM-5/ICD-10 criteria). A total of 283 questionnaires were distributed. After excluding 59 invalid responses (due to patterned answering or missing key information), 224 valid questionnaires were retained, resulting in an effective response rate of 79.15%. Based on similar models and variables in previous studies, the sample size of this research meets the requirements (Schoemann et al., 2017). The specific demographic characteristics are presented in Table 1.

### 2.2. Materials

This study employed four research instruments: the Dirty Dozen Dark Triad Measure, the Adolescent Self-Harm scale, the Adolescent Self-Rating Life Events Checklist, and the Center for Epidemiologic Studies Depression Scale. All scales used in this study are freely available for research purposes.

We conducted confirmatory factor analyses (CFA) to assess the structural validity of each scale. Fit indices included *χ*^2^/*df*, CFI, TLI, RMSEA, and SRMR, with cutoffs following conventional criteria (CFI/TLI > 0.90, RMSEA < 0.08, SRMR < 0.08).

#### 2.2.1. The Dirty Dozen Dark Triad Measure

The Dirty Dozen Dark Triad Measure used in this study was revised by Geng et al. (2015). The scale consists of 12 items measuring three dimensions: narcissism, psychopathy, and Machiavellianism, using a 7-point Likert scale ranging from 1 (“completely disagree”) to 7 (“completely agree”). In this study, the overall Cronbach’s α coefficient for the scale was 0.89, with coefficients for the three subscales ranging from 0.79 to 0.91. The results of Confirmatory factor analysis are presented in Table 2.

#### 2.2.2. The Adolescent Self—Rating Life Events Checklist

The Adolescent Self—Rating Life Events Checklist revised by X. Liu et al. (1997), was utilized in this study. This 8-item unidimensional scale employs a 5-point Likert format ranging from 1 (“never”) to 5 (“always”). In this study, the overall Cronbach’s α coefficient for the scale was 0.88. The results of Confirmatory factor analysis are presented in Table 2.

#### 2.2.3. Center for Epidemiologic Studies Depression Scale

The Center for Epidemiologic Studies Depression Scale used in this study was revised by X. C. Liu et al. (1995). This 20-item scale consists of four dimensions and employs a 4-point Likert scale ranging from 0 (“rarely or none of the time”) to 3 (“most or all of the time”), with four reverse-scored items. The total score ranges from 0 to 60. In the current study, the scale demonstrated good reliability, with an overall Cronbach’s α coefficient of 0.90 and subscale coefficients ranging from 0.71 to 0.91. The results of Confirmatory factor analysis are presented in Table 2.

#### 2.2.4. Adolescent Self-Injury Scale

The Adolescent Self-Harm Scale used in this study was revised by Feng (2008). This 18-item unidimensional scale employs a 4-point scoring system ranging from 1 (“0 times”) to 4 (“5 times or more”). The scale demonstrated excellent internal consistency in this study with an overall Cronbach’s α coefficient of 0.96. The results of Confirmatory factor analysis are presented in Table 2.

### 2.3. Research Procedures and Data Analysis

This study selected Year 7 to Year 9 Left-Behind Adolescents from three secondary schools in Inner Mongolia from 2024 to 2025 as research subjects, primarily based on the following two considerations: Firstly, the region is undergoing a transition from traditional nomadic culture to modern urbanisation. This social transformation has led to significant labour migration, resulting in a substantial population of left-behind adolescents. This unique social context provides a representative research setting for exploring the association between left-behind experiences and non-suicidal self-injury, facilitating deeper understanding of how specific sociocultural factors influence adolescents’ psychological and behavioural development. Secondly, confining the sample to three secondary schools within the same administrative and cultural region allows for greater control over variables influenced by macro-level geographical differences. This enhances homogeneity within the study group, thereby increasing the explanatory power and inferential value of research findings within comparable socio-cultural contexts.

After obtaining consent from students’ parents, teachers, and school administration, trained researchers administered the questionnaires collectively, with participants completing all measures within a designated time period. For data analysis, SPSS 27.0 was first used to perform descriptive statistical analysis, correlation analysis, and common method bias testing. Subsequently, Hayes’ PROCESS macro for SPSS was employed to examine the mediation model (using Model 4) and the moderated mediation model (using Model 59). All statistical analyses were conducted following standard procedures to ensure methodological rigor. This study has been approved by the Ethics Committee of Inner Mongolia Normal University (No. XL2024083001).

## 3. Results

### 3.1. Common Method Bias Test

The data collection in this study employed self-report measures, which may potentially introduce common method bias. To assess this possibility, Harman’s single-factor test was conducted. The results revealed 11 factors with eigenvalues greater than 1, with the first factor accounting for 29.72% of the total variance (<40%). These findings indicate that no serious common method bias was present in the study (Zhou & Long, 2004). Second, we performed a confirmatory factor analysis (CFA) comparing a single-factor model with our proposed measurement model. The results demonstrated poor fit for the single-factor model, whereas the hypothesized multi-factor model showed significantly better fit. Thus, both methods collectively suggest that serious common method bias is unlikely to be a concern in this study.

### 3.2. Descriptive Statistics and Correlation Analysis

The means, standard deviations, and correlation coefficients for all variables are shown in Table 3. Significant positive correlations were found among all variables, with correlation coefficients ranging from 0.22 to 0.54 (all *p* < 0.01).

### 3.3. Comparison of Scale Scores Between Male and Female Adolescents with Left-Behind Experience

Significant differences were observed in DS Total Score and NSSI Total Score by gender, with female adolescents with left-behind experiences scoring higher than male adolescents with left-behind experiences (see Table 4).

### 3.4. The Relationship Between the Dark Triad Traits and Non-Suicidal Self-Injury: Testing the Mediating Role of Negative Life Events

The analysis was conducted using Model 4 of Hayes’ PROCESS macro for SPSS with standardized variables, while controlling for demographic variables such as age and gender (Huang et al., 2024). The results demonstrated that: the dark triad traits positively predicted non-suicidal self-injury (NSSI) behaviors, negative life events positively predicted NSSI behaviors, and negative life events partially mediated the relationship between the dark triad traits and NSSI behaviors (see Table 5).

### 3.5. The Relationship Between the Dark Triad Traits and Non-Suicidal Self-Injury: A Moderated Mediation Model

The analysis was performed using Model 59 of Hayes’ PROCESS macro for SPSS with standardized variables, while controlling for demographic variables including age and gender (Huang et al., 2024). As shown in Table 6: First, the dark triad traits positively predicted negative life events (*β* = 0.15, *p* > 0.01), but the interaction term between the dark triad traits and depression did not significantly predict negative life events (*β* = 0.06, *p* > 0.01). This indicates that the direct effect of the dark triad traits on negative life events was not moderated by depression. Second, the dark triad traits positively predicted non-suicidal self-injury (NSSI) behaviors (*β* = 0.15, *p* > 0.01), while the interaction between the dark triad traits and depression did not significantly predict NSSI behaviors (*β* = 0.10, *p* > 0.01). Thus, the direct effect of the dark triad traits on NSSI was not moderated by depression. Third, negative life events positively predicted NSSI behaviors (*β* = 0.09, *p* < 0.001), and the interaction between negative life events and depression significantly predicted NSSI behaviors (*β* = 0.14, *p* < 0.01). Combined with previous findings, these results demonstrate that the latter half of the mediation pathway where the dark triad traits influences NSSI through negative life events—was moderated by depression.

**Figure 1:**
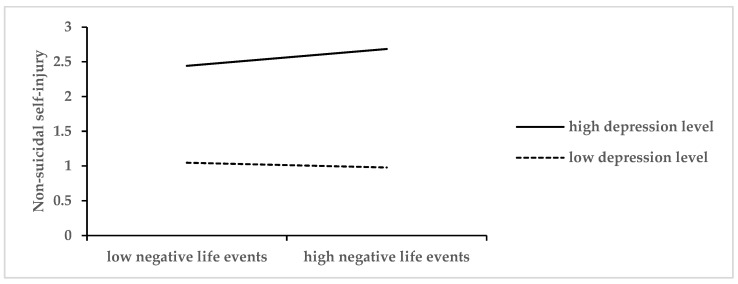
Simple Slope Analysis of Depression’s Moderating Effect Between Negative Life Events and Non-Suicidal Self-Injury (NSSI).

The study further examined depression’s moderating effect in the relationship between the dark triad traits and non-suicidal self-injury by conducting simple slope analysis with depression divided into high (*M* + 1*SD*) and low (*M* − 1*SD*) groups. The results, presented in Figure 1, revealed that among all pathways linking the dark triad traits to NSSI through negative life events, only the latter half of the mediation pathway was moderated by depression. Specifically, negative life events showed no significant predictive effect on NSSI behaviors at low depression levels (*β* = −0.05, *t* = −0.72, *p* > 0.01), but demonstrated a significant positive predictive effect at high depression levels (*β* = 0.20, *t* = 3.54, *p* < 0.001). These findings indicate that depression significantly moderates the impact of negative life events on NSSI, while not influencing the earlier pathway from the dark triad traits to negative life events.

## 4. Discussion

Previous studies have indicated that gender and age are significant factors influencing non-suicidal self-injury among adolescents (Xu et al., 2022). Consequently, this research incorporated both variables as control factors to mitigate potential confounding effects and enhance the reliability of findings. The analysis further confirmed significant gender differences in depression and non-suicidal self-injury scores, with female adolescents exhibiting higher total depression scores and higher total non-suicidal self-injury scores than their male counterparts. This disparity may stem from several contributing factors: Firstly, in terms of emotional traits, females typically exhibit greater emotional sensitivity and internalising tendencies. When confronting stress or adverse environments, they often experience more profound distress, rendering them more susceptible to depressive states and inclined to direct suffering inward, manifesting as non-suicidal self-injury (Fischer et al., 2018). Secondly, in terms of coping strategies, males frequently channel emotions through physical activities (such as sports or competitive pursuits), whereas females more commonly adopt introverted, self-directed approaches (Ovsyanik et al., 2022). Self-harm may serve as a means of releasing accumulated pressure, which, when prolonged, exacerbates depressive symptoms.

The dark triad traits significantly and positively predicted non-suicidal self-injury (NSSI) among left-behind adolescents, confirming Hypothesis H1 of this study. This finding not only aligns with previous research but also supports the Dual-Harm Cognitive-Affective Model’s proposition that adolescent NSSI results from the interaction between proximal and distal factors (Shafti et al., 2021; P. P. Li, 2020). Several mechanisms may explain this relationship. Developmentally, adolescents’ immature physical and psychological growth often manifests in characteristics like arrogance, grandiose fantasies, excessive vanity, and strong power-seeking tendencies (Paulhus & Williams, 2002). These traits frequently trigger peer rejection. When such individuals eventually recognize their social undesirability and develop low self-esteem (yet lack capacity for outward aggression) they may turn to self-directed injurious behaviors as an alternative. Furthermore, compared to other populations, adolescents face compounded pressures from parental expectations, peer relationships, and academic demands. When lacking adequate coping strategies to resolve these challenges, some individuals resort to NSSI as a maladaptive means of obtaining temporary relief and escaping reality. This pattern appears particularly pronounced among left-behind adolescents, whose parental absence deprives them of critical emotional support during this vulnerable developmental period.

Negative life events serve as an intrinsic mediating mechanism linking the dark triad traits to non-suicidal self-injury (NSSI) in adolescents, which validates Hypothesis H2 of this study and supports the perspective of the Stress-Diathesis Model (Mann et al., 1999; Zhao & Liu, 2025). Individuals with dark triad traits are more sensitive to life stimuli and more prone to interpreting them as negative life events (Zhao & Liu, 2025). Specifically, although adolescents have reached the formal operational stage of cognitive development and theoretically possess the ability to consider others’ perspectives, real-life situational and emotional factors often hinder this capacity. In social interactions, even mundane events may be perceived as personally harmful, leading to self-injurious behaviors as an outlet for their distress. Additionally, academic pressure, interpersonal relationships, and physical appearance are significant triggers of negative life events. Compared to elementary school children, adolescents face greater academic demands, enduring not only heavy coursework but also intense scrutiny and pressure from schools, teachers, and parents. Under such multifaceted stress, they are highly susceptible to psychological issues, resorting to self-injury as a coping mechanism. Moreover, adolescents remain psychologically immature, and experiences like friendship breakdowns or peer bullying can severely disrupt their lives. Coupled with their heightened sensitivity, fragility, and reluctance to seek help due to strong self-esteem, they often resort to unhealthy methods to process negative emotions. Lastly, adolescents increasingly focus on their physical appearance, tending to exaggerate minor flaws, which fosters feelings of inferiority. This sense of inadequacy further permeates various aspects of their lives, adversely affecting their daily functioning and prompting harmful behaviors. In summary, negative life events not only affect the physical development of adolescents but also adversely impact their psychological growth and social adaptation. Previous studies have shown that the incidence of suicidal behavior is relatively high among adolescents with depression, and negative life events such as school bullying, suicidal ideation, emotional abuse, and poor interpersonal relationships are significant risk factors for suicidal behavior (Gao et al., 2024). Therefore, parents, teachers, and schools should make concerted efforts to shield adolescents from the trauma caused by negative life events, thereby reducing the occurrence of maladaptive behaviors in this population.

In the mediating model examining how the dark triad traits influence adolescent non-suicidal self-injury (NSSI) through negative life events, depression was found to play a moderating role in two key pathways: the “dark triad traits → negative life events” path and the “dark triad traits → NSSI” path, partially confirming Hypothesis H3 of this study. Regarding the moderating effect of depression on the relationship between the dark triad traits and negative life events, adolescents with higher depressive symptoms perceived greater psychological trauma from negative life events compared to those with lower depression levels. This effect can be explained through two mechanisms. Cognitively, depression biases individuals toward negative interpretations, leading them to perceive life experiences through a pessimistic lens. Behaviorally, highly depressed adolescents exhibit blunted reward sensitivity and motivational avoidance (W. Liu et al., 2024). Even when parents, teachers, or peers attempt positive interactions, these individuals struggle to respond with emotional engagement, reinforcing a negative interpersonal cycle that exacerbates their sensitivity to adverse experiences. Furthermore, in the pathway where depression moderates the relationship between the dark triad traits and NSSI, the predictive effect of dark triad traits on self-injurious behaviors was significantly stronger among individuals with high depression levels. This finding aligns with the Frustration-Aggression theory. For individuals with low depressive symptoms, although dark triad traits may create developmental obstacles, their optimistic mindset helps mitigate these adverse effects, allowing them to adopt adaptive coping strategies when facing setbacks. In contrast, highly depressed adolescents lack such psychological resilience, making it difficult to overcome the persistent frustrations associated with dark triad traits. When encountering daily stressors, they are more prone to risky behaviors (e.g., substance abuse), self-harm, and even suicidal tendencies. This contrast highlights the critical role of depressive symptoms in amplifying the detrimental effects of dark triad traits on self-injurious behaviors (Pozuelo et al., 2022).

This study has several limitations. First, as a cross-sectional study, it cannot establish causal relationships between variables. Future research could employ longitudinal designs to further examine the relationship between the dark triad traits and adolescent non-suicidal self-injury (NSSI) behaviors, thereby exploring the underlying mechanisms behind this association. Meanwhile, since NSSI is a socially stigmatized behavior, questionnaire-based measurements may be subject to social desirability bias. Subsequent studies should incorporate alternative methods such as observational or experimental approaches to mitigate this potential bias. Moreover, as this study exclusively selected junior secondary students from three secondary schools in Inner Mongolia as its sample, its applicability is subject to certain limitations. The findings are primarily relevant to left-behind adolescents within China who share similar socio-cultural backgrounds—namely, young people residing in regions undergoing transition from traditional nomadic cultures to modern urbanisation, characterised by significant labour outflow. In addition, while this study focused specifically on the influence of dark triad traits on adolescent NSSI, future investigations should expand to examine whether other personality types similarly affect NSSI behaviors. Meanwhile, beyond the mediating role of negative life events and the moderating effect of depression in the relationship between dark triad traits and NSSI, whether other mediating or moderating mechanisms exist remains to be explored in future research.

## 5. Conclusions

The dark triad traits significantly and positively predict non-suicidal self-injury (NSSI) among adolescents with left-behind experiences. In addition, Negative life events play a mediating role in the relationship between the dark triad traits and NSSI in left-behind adolescents. Meanwhile, Depression moderates the latter half of the pathway “dark triad traits → negative life events → NSSI in left-behind adolescents.” Specifically, under high depression levels, negative life events significantly predict NSSI in left-behind adolescents. In contrast, under low depression levels, negative life events do not show a significant predictive effect on NSSI. Furthermore, this study carries several practical implications. First, implementing routine dark triad traits assessments in school mental health programs could help identify high-risk left-behind adolescents. Early psychological interventions targeting these personality factors may prevent NSSI development. Second, since depression activates the harmful impact of negative life events, mental health services for left-behind adolescents should prioritize depression treatment. This could disrupt the pathological pathway from adversity to self-injury.

## Figures and Tables

**Table 1:** Demographic characteristics (*n* = 224).

Category	Subgroup	Count	Percentage
Gender	Male	130	58.04%
	Female	91	40.63%
	Missing data	3	1.33%
Grade level	7th grade	127	56.70%
	8th grade	37	16.52%
	9th grade	34	15.18%
	Missing data	26	11.61%

**Table 2:** Results of Confirmatory Factor Analysis for Key Variables (*n* = 224).

	*χ*^2^/*df*	TLI	CFI	RMSEA	SRMR
DTT	2.70	0.96	0.98	0.04	0.04
NLE	4.25	0.95	0.97	0.06	0.03
DS	4.27	0.90	0.91	0.06	0.05
NSSI	4.85	0.93	0.94	0.06	0.04

Note: DTT = Dark Triad Traits; NLE = Negative Life Events; DS = Depressive Symptoms; NSSI = Non-Suicidal Self-Injury.

**Table 3:** Means, Standard Deviations, and Correlation Coefficients of Variables.

	*M* ± *SD*	DTT	NLE	DS	NSSI
AGE	14.30 ± 1.15				
DTT	2.27 ± 0.86	1			
NLE	2.31 ± 0.78	0.22 **	1		
DS	2.12 ± 0.55	0.28 **	0.27 **	1	
NSSI	1.31 ± 0.61	0.31 **	0.28 **	0.54 **	1

Note: ** *p* < 0.01. DTT = Dark Triad Traits; NLE = Negative Life Events; DS = Depressive Symptoms; NSSI = Non-Suicidal Self-Injury.

**Table 4:** Gender Difference Tests on Main Variables Among Left-Behind Adolescents.

Variable	Male (*n* = 130)	Female (*n* = 91)	*t*	*p*
DTT	26.60 ± 10.41	28.20 ± 9.83	−1.16	0.249
NLE	18.65 ± 6.46	18.44 ± 5.96	0.24	0.808
DS	36.06 ± 10.30	39.10 ± 11.61	−2.00 *	0.047
NSSI	22.26 ± 9.83	25.43 ± 12.11	−2.04 *	0.042

Note: * *p* < 0.05. DTT = Dark Triad Traits; NLE = Negative Life Events; DS = Depressive Symptoms; NSSI = Non-Suicidal Self-Injury.

**Table 5:** Test of the Mediation Model.

	NLE				NSSI			
	*β*	*t*	LLCI	ULCI	*β*	*t*	LLCI	ULCI
DTT	0.22	3.14 **	0.08	0.35	0.28	4.16 **	0.15	0.41
NLE					0.22	3.32 ***	0.09	0.35
*R* ^2^	0.08				0.17			
*F*	5.71 ***				10.67 ***			

Note: ** *p* < 0.01, *** *p* < 0.001. DTT = Dark Triad Traits; NLE = Negative Life Events; NSSI = Non-Suicidal Self-Injury.

**Table 6:** Test of the Moderated Mediation Model.

	NLE				NSSI			
	*β*	*t*	LLCI	ULCI	*β*	*t*	LLCI	ULCI
DTP (X)	0.15	2.08	0.01	0.29	0.15	2.53 **	0.03	0.27
D (U)	0.22	3.05 **	0.08	0.36	0.41	6.64 ***	0.29	0.54
X × U	0.06	1.10	−0.05	0.18	0.10	1.91	−0.01	0.19
NLE (M)					0.09	1.55 ***	−0.03	0.21
M × U					0.14	2.65 **	0.03	0.24
*R* ^2^	0.13				0.39			
*F*	6.10 ***				18.25 ***			

Note: ** *p* < 0.01, *** *p* < 0.001. NLE = Negative Life Events; NSSI = Non-Suicidal Self-Injury.

## Data Availability

The data supporting the conclusions of this article will be made available by the authors on request.
